# *Arabidopsis* Myosins XI1, XI2, and XIK Are Crucial for Gravity-Induced Bending of Inflorescence Stems

**DOI:** 10.3389/fpls.2016.01932

**Published:** 2016-12-21

**Authors:** Kristiina Talts, Birger Ilau, Eve-Ly Ojangu, Krista Tanner, Valera V. Peremyslov, Valerian V. Dolja, Erkki Truve, Heiti Paves

**Affiliations:** ^1^Department of Gene Technology, Tallinn University of TechnologyTallinn, Estonia; ^2^Department of Botany and Plant Pathology and Center for Genome Research and Biocomputing, Oregon State University, CorvallisOR, USA

**Keywords:** *Arabidopsis*, actin filaments, myosins, T-DNA mutants, gravitropism, amyloplasts

## Abstract

Myosins and actin filaments in the actomyosin system act in concert in regulating cell structure and dynamics and are also assumed to contribute to plant gravitropic response. To investigate the role of the actomyosin system in the inflorescence stem gravitropism, we used single and multiple mutants affecting each of the 17 *Arabidopsis* myosins of class VIII and XI. We show that class XI but not class VIII myosins are required for stem gravitropism. Simultaneous loss of function of myosins XI1, XI2, and XIK leads to impaired gravitropic bending that is correlated with altered growth, stiffness, and insufficient sedimentation of gravity sensing amyloplasts in stem endodermal cells. The gravitropic defect of the corresponding triple mutant *xi1 xi2 xik* could be rescued by stable expression of the functional XIK:YFP in the mutant background, indicating a role of class XI myosins in this process. Altogether, our results emphasize the critical contributions of myosins XI in stem gravitropism of *Arabidopsis*.

## Introduction

Myosins are a family of eukaryotic motor proteins with conserved structure that perform multiple functions in cell organization and motility. They use energy derived from ATP hydrolysis to slide along actin filaments. Plants have two classes of myosins: class VIII and XI, containing 4 and 13 genes in *Arabidopsis*, respectively (Reddy and Day, 2001; Peremyslov et al., 2011). Class VIII myosins were reported in association with plasmodesmata, endosomes, endoplasmic reticulum (ER), plasma membrane of newly formed cell walls, and plastids (Reichelt et al., 1999; Avisar et al., 2008a; Golomb et al., 2008; Sattarzadeh et al., 2008; Haraguchi et al., 2014; Wu and Bezanilla, 2014) although their exact functions remain largely enigmatic.

Studies on myosin XI have shown that these myosins drive the motility of different organelles and vesicles (Avisar et al., 2008b, 2009; Peremyslov et al., 2008, 2010, 2013; Prokhnevsky et al., 2008; Sparkes et al., 2008; Ueda et al., 2010; Tamura et al., 2013), cytoplasmic streaming (Shimmen and Yokota, 2004; Avisar et al., 2012; Tominaga et al., 2013; Peremyslov et al., 2015), cell expansion and plant growth (Ojangu et al., 2007, 2012; Peremyslov et al., 2008, 2010; Prokhnevsky et al., 2008; Park and Nebenführ, 2013; Madison et al., 2015; Okamoto et al., 2015). For example, *Arabidopsis* myosins XI1, XI2, XIB, XIC, XIE, XIF, XIG, XII, and XIK have been reported to have overlapping roles in pollen tube and root hair elongation, trichome development, plant size, and organelle motility (Prokhnevsky et al., 2008; Peremyslov et al., 2010; Ojangu et al., 2012; Madison et al., 2015; Okamoto et al., 2015). Triple mutant *xi1 xi2 xik* exhibits reduced fertility and decreased growth of epidermal cells affecting overall plant size (Peremyslov et al., 2010; Ojangu et al., 2012). In addition, it has been shown that myosins regulate dynamics of actin filaments and bundles. Loss of myosins leads to reshaping of longitudinal F-actin cables into randomly and more transversely oriented ones making the cytoskeleton less dynamic (Peremyslov et al., 2010; Ueda et al., 2010; Vidali et al., 2010; Cai et al., 2014; Madison et al., 2015).

Actin filaments have also been proposed to be one of the components in gravity sensing (Blancaflor, 2013). Gravitropism is the ability of land plants to respond to the direction of gravity and reorient organs accordingly. Gravity stimulus is perceived in gravity-sensing cells called statocytes localized to endodermis in shoots and to innermost columella cells in the root cap (Sack, 1991; Fukaki et al., 1998; Morita and Tasaka, 2004). Statocytes contain starch-filled amyloplasts that act as statoliths: they sense the direction of gravity and translocate along the gravity vector (Morita and Tasaka, 2004). Biochemical signals are transmitted to responding tissues where asymmetric cell growth takes place: shoots curve away from the gravity vector and roots grow toward the gravity vector (Kiss, 2000; Valster and Blancaflor, 2007; Gilroy and Masson, 2008).

The role of actomyosin system in gravitropism is starting to be revealed. Actin filaments have been reported to interact with gravity sensitive amyloplasts (Saito et al., 2005; Nakamura et al., 2011). Inactivation of *Arabidopsis* RING-type E3 ligase, SGR9, which is localized to endodermal amyloplasts, had reduced stem gravitropism and defective amyloplast sedimentation because of clusters of amyloplasts being entangled with actin filaments. SGR9 was proposed to function in shoot gravitropism by modulating the interaction between the amyloplasts and actin filaments and promoting their detachment from actin filaments (Nakamura et al., 2011). The findings of Zhang et al. (2011) indicated that in the endodermal cells of reoriented cut snapdragon spikes amyloplasts were surrounded by and connected to actin filaments through myosin-like proteins. Moreover, *Arabidopsis* myosins XIF and XIK were demonstrated to regulate organ straightening in gravitropism (Okamoto et al., 2015). It was also found that simultaneous inactivation of myosins XI and their cognate vesicular MyoB receptors results in bended stems, siliques, and roots (Peremyslov et al., 2015).

In this study, we investigated the role of myosin family in gravitropic bending. We used T-DNA insertional mutants for all 17 myosin genes to characterize gravitropic response in *Arabidopsis* inflorescence stems of single mutants, previously characterized double mutants *xi1 xi2, xi1 xik, xi2 xik* and class XI triple mutant *xi1 xi2 xik* (Ojangu et al., 2012), as well as newly generated double mutant *xih xik*, triple mutant *xi2 xik xih* and class VIII quadruple mutant *viii1 viii2 viiia viiib*. Because class XI triple mutant *xi1 xi2 xik* showed impaired gravitropic response, it was analyzed further for physical features, the actin cytoskeleton and sedimentation of amyloplasts. We show that myosins XI are involved in stem gravitropism and discuss possible reasons underlying this phenotype.

## Materials and Methods

### Plant Material and Growth Conditions

Seeds of single T-DNA insertion lines of *Arabidopsis thaliana* ecotype Columbia-0 (Col-0) were obtained from the Nottingham Arabidopsis Stock Centre. The T-DNA insertion lines for the myosin genes are listed in Supplementary Table S1. Double mutants *xi1 xi2, xi1 xik, xi2 xik*, triple mutant *xi1 xi2 xik* and triple mutant transformed with the gene encoding YFP-tagged myosin XIK (*xi1 xi2 xik XIK:YFP*) were described earlier (Ojangu et al., 2012; Peremyslov et al., 2012). To generate *xih xik*, triple mutant *xi2 xik xih* and quadruple mutant *viii1 viii2 viiia viiib*, homozygous lines were crossed and selected by PCR screening (Solis BioDyne).

Cold stratified seeds were held in water at 4°C for 1 day before sowing in the soil containing 50% (v/v) vermiculite. Plants were grown in growth chambers under 16 h light/8 h dark period at 22 ± 2°C and 60% of relative humidity.

For the analysis of myosin mRNA levels in T-DNA mutants, *Arabidopsis* (Col-0) wild type and T-DNA mutant seeds were surface sterilized and grown on 0.5× MS medium (Murashige and Skoog, 1962) in growth chambers as described above.

### RNA Extraction and Reverse Transcription – Quantitative Real-Time PCR

Total RNA was isolated from 100 mg of plant material according to the method described by Oñate-Sánchez and Vicente-Carbajosa (2008). Buffer volumes were scaled up three times. For the analysis of myosin mRNA levels in T-DNA mutants, RNA of 7-day-old seedlings was extracted. For the analysis of the effect of gravistimulation on myosin mRNA expression, RNA was extracted from whole stems with cauline leaves and flowers. Eight micrograms of extracted RNA was treated with RNase-free DNase I (Thermo Scientific). cDNA was synthesized from 5 μg of DNase-treated RNA using Maxima Reverse Transcriptase (Thermo Scientific) and random hexamer primer. cDNAs were diluted twofold for qPCR. All reverse transcription – quantitative real-time PCR (RT-qPCR) reactions were performed in 384-well plates on the LightCycler 480 instrument (Roche Applied Science). qPCR reactions were performed in duplicate and Cq values were averaged. Each 7 μl reaction contained 1.4 μl 5× HOT FIREPol^®^ EvaGreen^®^ qPCR Mix Plus (no ROX) (Solis Biodyne), 0.7 μl diluted cDNA and 3.5 pmol each primer. qPCR conditions were as follows: initial denaturation at 95°C for 12 min, followed by 45 cycles of 95°C for 15 s, 59°C for 30 s, and 72°C for 30 s. Primers used for qPCR experiments were designed to anneal downstream of T-DNA insertions at the 3′ end of each gene and are listed in Supplementary Table S2. Primers for reference genes were chosen according to Czechowski et al. (2005).

To quantify myosin mRNA in insertion lines, targets were normalized to SAND mRNA levels. Three experiments were performed with each mutant and depending on the mutant there was one or two biological replicates per experiment. ΔΔCq calculation method was used to calculate relative differences in mRNA levels between mutant and wild type. Fold changes from independent experiments were subjected to log-transformation, global mean centering and autoscaling as described by Willems et al. (2008). Statistical analysis was performed with JMP 12.2.0 software, either *t*-test or ANOVA with Dunnett’s *post hoc* comparison was used, depending on whether one or more groups were compared to wild type.

In gravistimulation experiments, four reference genes were used for normalization: SAND, UBC, expressed sequence EX70 and PP2A subunit PDF2 (Czechowski et al., 2005; Supplementary Table S2). Three independent experiments with four biological replicates in each were performed. Reference gene stability was analyzed using GeNorm M and coefficient of variation in qbase^PLUS^ software (Hellemans et al., 2007). Geometric mean of relative quantities of four reference mRNAs was used to calculate the normalization factors. Normalized relative quantities were log transformed, mean centered, and autoscaled (Willems et al., 2008). Statistical analysis was performed with JMP 12.2.0 software, *t*-test was used to calculate two-tailed *p*-values.

### Gravitropism Assay of Inflorescence Stems

Primary inflorescence stems of 8–10 cm were cut from 5- to 6-week-old plants with a razor blade and placed into water containing 1.5 ml Eppendorf tubes through a hole in the cap which was sealed with 4% (w/v) low melting point agarose (Thermo Scientific) solution to avoid whirling of the stems. Stems were placed into dark chamber using vertical racks that kept the tubes and stems in horizontal positions. Gravitropic responses of *Arabidopsis* stems were recorded in the dark chamber using Nikon D7000 SLR camera with built-in flash. A total of 600 frames with 1 min intervals were taken during each experiment. The series of pictures were imported to ImageJ (Fiji) software (Schindelin et al., 2012) and saved as .avi files. Gravitropic curvature was depicted as sequence of angles between apical part of the stem and horizontal base line during gravistimulation. For the calculation of relative average speed, movements of stem tips were tracked using ImageJ Manual Tracking plug-in. Changes of stem tip position were measured in vertical and horizontal direction and absolute values of the changes were calculated using Pythagorean theorem. Positive and negative values were given according to movement of stem tip upward/toward the base and downward/away from the base, correspondingly. Relative average speed was calculated between the time points of minimum and maximum curvature value. Growth rate of stems was measured by changes in stem elongation during 600 min.

### Histological Sections of Inflorescence Stems

Stem segments of gravisensitive region from 5- to 6-week-old inflorescences (8–10 cm) were embedded in Spurr resin (Spurr, 1969). Segments were fixed in 2.8% glutaraldehyde in 0.1 M HEPES buffer with 0.01% (v/v) Triton X100 for 2 h at room temperature and left at 4°C overnight. Followed by three times of washing for 15 min with the same buffer and dehydration in graded ethanol series (30, 40, 50, 60, 70, 80, and 90%) and then twice in absolute acetone, each step for 20 min. Infiltration was done on rotator in graded acetone/resin series 3:1, 1:1, 1:3 (v/v) for 6, 8, and 16 h, respectively, followed by two to three changes of pure Spurr resin. Stem segments were transferred to embedding molds and left to polymerize at 60°C for 2 days. One micrometer thick sections were cut with microtome (PowerTome PT-XL, RMC Boeckeler), placed on slides, stained with 1% (w/v) toluidine blue O for 10 min on 60°C hotplate and rinsed with deionized water.

Sections were visualized using Olympus BX61 microscope (20 × 0.75 NA and 40 × 0.90 NA).

### Measurement of Mechanical Properties

Freshly cut primary inflorescence stems were subjected to 3-point flexure test by Instron 5866 testing system. 2 cm segment was excised from gravisensitive region of the stem and placed horizontally on two stationary custom-made supports at the distance of 10 mm. The load cell of 2.5 N was applied centrally at the speed of 20 mm/min and continuous measurements of stress and strain were made simultaneously for increments of load. Flexural modulus of elasticity was calculated according to Johnson et al. (2003) as follows:

$$\begin{array}{c} E_{f}=\frac{4}{3}\times \frac{P}{\delta }\times \frac{L^{3}}{\pi d^{4}} \end{array}$$

P—load applied to the stem segment; δ—corresponding deflection at the point of load application; (P/δ)—the gradient of the initial straight-line portion of the load-deflection curve; L—length between supports; d—diameter of the stem segment. Stem diameters were measured with digital micrometer.

### Visualization of Actin Filaments

For visualization of actin filaments, wild type and *xi1 xi2 xik* mutant plants were transformed by floral dip method (Clough and Bent, 1998) to stably express the construct containing a single GFP tag at the N- and C-termini of actin-binding domain 2 from *Arabidopsis* fimbrin 1 under the control of the CaMV 35S promoter (35S::GFP-fABD2-GFP) (Wang et al., 2008). Stem segment of 1 cm was excised from the region of 1–2 cm below the apex, hand-cut longitudinally on the glass slide with razor blade and immediately immersed in 95% perfluorodecalin (Sigma-Aldrich). Silicon spacer was applied between glass slide and cover slip to prevent crushing stem segments. GFP-fABD2-GFP was imaged with Carl Zeiss LSM 510 META confocal laser scanning microscope using water immersion objective (63 × 1.2 NA), excitation at 488 nm. GFP fluorescence was detected with 505–550 nm band-pass filter.

### Actin Filament Analysis

Z-stacks of confocal images were combined into single image by maximal intensity projections. Average angles of actin filaments were measured against longitudinal axis of the cell and parallelness with respect to each other according to the methods of Ueda et al. (2010).

### Amyloplast Sedimentation

Six to eight centimeters primary inflorescence stems were cut and placed into water containing 1.5 ml Eppendorf tubes through a hole in the cap which was sealed with 4% (w/v) agarose solution. Stems were placed in darkness and gravistimulated by turning them upside down for 10, 20, and 40 min. The region 1–2 cm from the apex was excised and fixed with direction of gravity maintained constant in Carnoy fixative (3:1, 96% ethanol:acetic acid) overnight and embedded in Steedman’s wax (Vitha et al., 2000). For infiltration, segments were first incubated in 96% ethanol twice for 1–2 h at room temperature, then in graded ethanol/wax series (2:1, 1:1, 1:2 v/v) at 37°C, 1 h each, followed by pure wax twice for 1.5 h. Stem segments were transferred to embedding molds and left to polymerize at room temperature overnight. Longitudinal 16 μm thick sections were cut with a microtome (Ergostar HM 200, Microm International) and placed on slides. Slides were dewaxed in 96% ethanol twice for 30–40 min and rehydrated in graded ethanol series (70, 50, and 30%), 20 min each. Samples were stained for starch with IKI (2.5% v/v iodine, 2% w/v potassium iodide) and visualized using Olympus BX61 microscope (40 × 0.90 NA).

## Results

### Selection of T-DNA Mutant Lines for Gravitropism Assay

**FIGURE 1:**
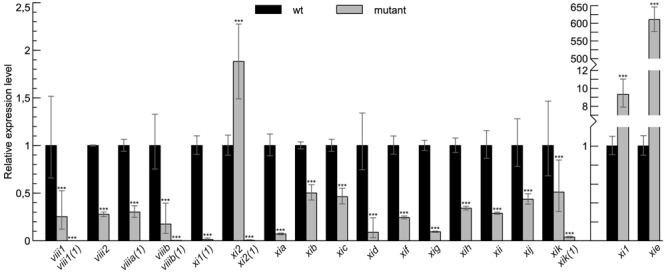
**Myosin mRNA levels in T-DNA mutant lines used in this study.** RT-qPCR analysis of myosin mRNA levels in myosin T-DNA mutants relative to wild type (wt) control (set to 1). Error bars represent 95% confidence intervals (CI), *n* = 3–6; ^∗∗∗^*p* < 0.001.

To analyze the role of myosins in gravity response of the inflorescence stems of *Arabidopsis*, we performed a gravitropism assay on myosin single, double, triple, and quadruple mutant lines. First, we analyzed myosin mRNA content of each homozygous T-DNA mutant line by reverse transcription followed by real-time PCR (RT-qPCR). Only single mutant lines where RT-qPCR showed the most down-regulation of expression were selected for gravitropism assay: *viii1(1), viii2, viiia(1), viiib(1), xi1(1), xi2(1), xia, xib, xic, xid, xif, xig, xih, xii, xij*, and *xik(1)*. The only exception was *xie* for which no downregulated allele was found (**Figure 1**). Because double mutants *xi1 xi2, xi1 xik, xi2 xik, xih xik*, triple mutants *xi1 xi2 xik* and *xi2 xik xih* and quadruple mutant *viii1 viii2 viiia viiib* were made previously using different single mutant lines, the expression levels of myosins in these lines were also measured and confirmed to be reduced in mutants *viii1, viiib*, and *xik* and increased in *xi1* and *xi2* (**Figure 1**).

### Stem Gravitropism is Altered in Myosin XI1, XI2, and XIK Deficient Plants

**FIGURE 2:**
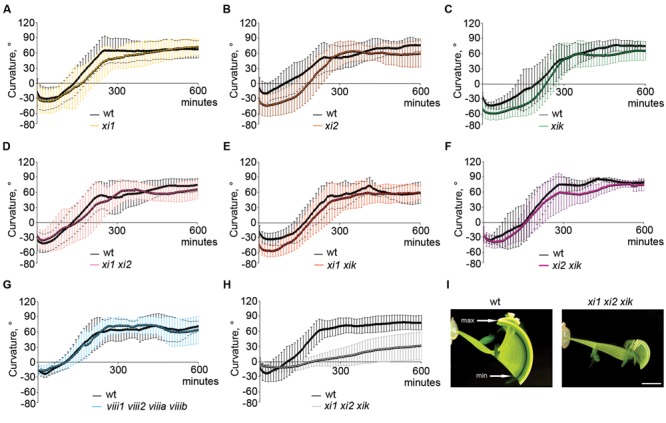
**Gravitropic response of 5-week-old *Arabidopsis* inflorescence stems during 10 h of gravistimulation. (A–H)** Time course of gravitropic responses of wild type (wt) and **(A–C)** single, **(D–F)** double, **(G)** quadruple, and **(H)** triple mutants. Error bars represent SD, *n* = 6–20. **(I)** Gravitropic phenotype of wild type and *xi1 xi2 xik.* Overlay of 600 images taken with 1 min intervals. min indicates the time point when subsiding of the stem tips changes to ascension, max shows the highest point of gravitropic curvature. Time between min and max was the basis for calculation of average speed. Scale bar 10 mm.

**FIGURE 3:**
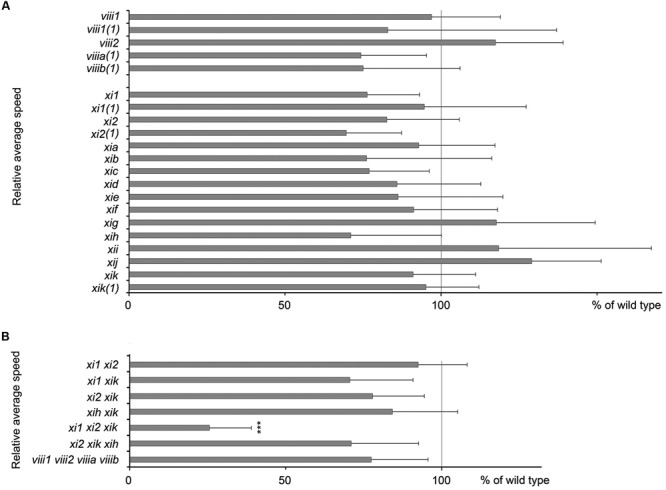
**Average speed of inflorescence stems tip movements during gravistimulation. (A)** Relative average speed of inflorescence stems of myosin single mutants and **(B)** double, triple, and quadruple mutants. Average speed is expressed as a percentage of wild type average speed (set to 100%). For every mutant line individual experiment was done together with wild type control. Error bars represent 95% CI, *n* = 21; ^∗∗∗^*p* < 0.001 (Student’s *t*-test).

For gravitropism assay, inflorescence stems were placed horizontally and gravitropic response was characterized by gravitropic curvature and relative average speed. All stems of single mutants, double mutants, triple mutant *xi2 xik xih* and quadruple mutant *viii1 viii2 viiia viiib* bent up to the same extent as wild type stems (**Figures 2A–G** and data not shown) and at an average speed comparable to that of wild type (**Figure 3**). The gravitropic response of *xi1 xi2 xik* was dramatically different resulting in delay of bending and shallower curvature compared to wild type (**Figures 2H,I**). While stems of wild type started to curve up within 30 min, continued bending and reached near vertical steady state, the stems of *xi1 xi2 xik* started to curve up only within 130 min and did not reach the maximum curvature value of the wild type even after 10 h of gravistimulation (**Figure 2H**). The average translocation speed in triple mutant was fourfold less than that in the wild type (**Figure 3B**). These results showed clearly that class XI but not class VIII myosins are required for stem gravitropism.

**FIGURE 4:**
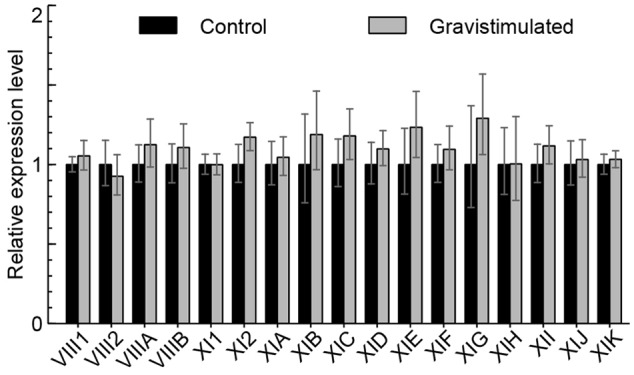
**Myosin mRNA levels after 4 h of gravistimulation.** RT-qPCR analysis of myosin mRNA levels in gravistimulated wild type plants relative to unstimulated control (set to 1). Error bars represent 95% CI, *n* = 12.

The expression of myosins in wild type inflorescence stems gravistimulated for 4 h compared to non-gravistimulated wild type stems was also analyzed. **Figure 4** shows that there is no difference in the expression of myosins during gravistimulation relative to the control, indicating that myosins are not transcriptionally regulated by gravitropic bending.

### *xi1 xi2 xik* Exhibits Reduced Growth Rate and More Rigid Inflorescence Stem

**FIGURE 5:**
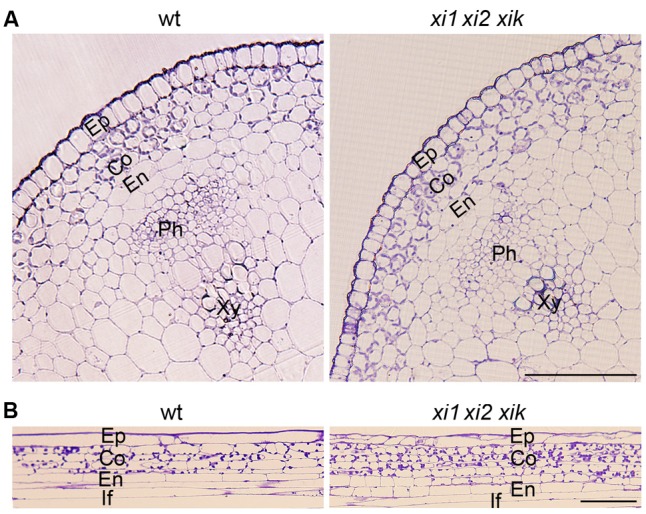
**Morphology of wild type and *xi1 xi2 xik* gravisensitive region. (A)** Cross-sections of inflorescence stems. **(B)** Longitudinal sections of inflorescence stems. Toluidine blue staining. Ep, epidermis; Co, cortex; En, endodermis; Ph, phloem; Xy, xylem; If, interfascicular fiber. Scale bars 50 μm.

Delayed and insufficient gravitropic response of *xi1 xi2 xik* implied that the physical features of the stem might be impaired. For analysis of stem morphology, we made cross sections and longitudinal sections of the stem’s gravisensitive region and found that radial cell organization of triple mutant was not different from that of the wild type. Inflorescence stems had one epidermal cell layer, three or four cortex layers, and one endodermal cell layer (**Figure 5**).

**FIGURE 6:**
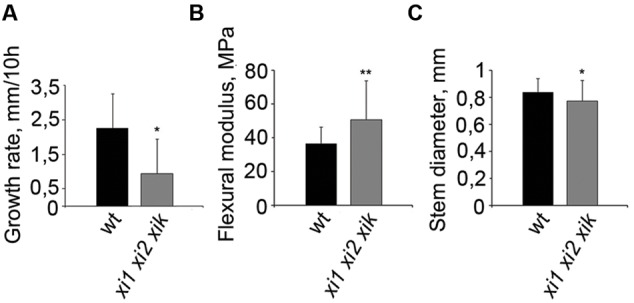
**Physical properties of wild type and *xi1 xi2 xik* inflorescence stems. (A)** Growth rates of inflorescence stems during 10-h gravitropism. Error bars represent SD, *n* = 9; ^∗^*p* < 0.05 (Student’s *t*-test). **(B)** Tendency for stems to bend in gravisensitive region according to flexure test. Higher flexural modulus of triple mutant shows greater stiffness of the stem. **(C)** Diameters of the same stems assayed in flexure test. Error bars represent SD, *n* = 35–45; ^∗^*p* < 0.05; ^∗∗^*p* < 0.01 (Student’s *t*-test).

As gravitropism requires organ growth and triple mutant has been shown to have shorter inflorescence stems than wild type (Peremyslov et al., 2010; Ojangu et al., 2012), we measured the elongation rates of stems during our 10-h gravitropism experiments and found it to be about 50% of that of wild type in triple mutant (**Figure 6A**). Next we performed flexure test for measuring stiffness of gravisensitive region. In flexure test we calculated flexural modulus of elasticity which shows the stiffness of the stems at the load applied. Compared to the wild type, *xi1 xi2 xik* had a 28% increase in bending stiffness and slightly reduced diameter (**Figures 6B,C**). These results suggest that myosins XI1, XI2, and XIK play a role in growth and rigidity formation of the inflorescence stem that likely affect gravitropic bending.

### Organization of Actin Filaments in *xi1 xi2 xik* Endodermal Cells is Not Altered

**FIGURE 7:**
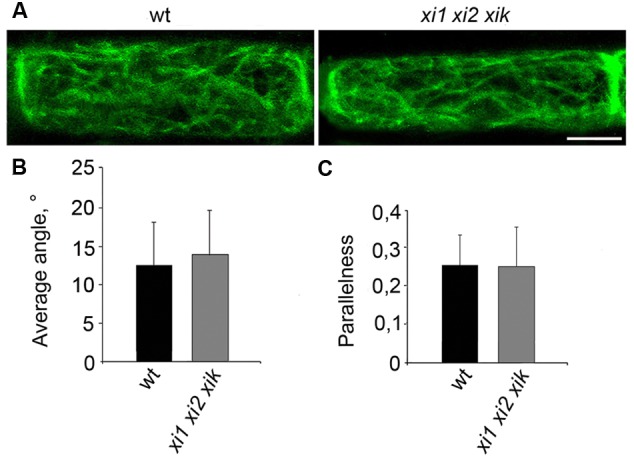
**Organization of actin filaments in living endodermal cells. (A)** GFP-fABD-GFP-labeled actin filaments in wild type (wt) and *xi1 xi2 xik* endodermal cells. **(B)** Average angle of actin filaments with respect to longitudinal axis of the cell. **(C)** Parallelness of actin filaments with respect to each other. Error bars represent SD, *n* = 28–29. Scale bar 20 μm.

It is known that epidermal cells of triple mutant *xi1 xi2 xik* have alterations in the architecture of actin filaments (Peremyslov et al., 2010; Ueda et al., 2010; Cai et al., 2014). Therefore we analyzed F-actin organization in living endodermal cells expressing GFP-fABD-GFP (actin-binding domain 2 from *Arabidopsis* fimbrin 1) F-actin marker in the wild type and *xi1 xi2 xik* stems. Confocal microscopy revealed extensive network of actin filament bundles in both wild type and triple mutant endodermal cells without any apparent differences in their organization (**Figure 7A**). To analyze F-actin organization quantitatively, we measured average angles against the longitudinal axis of the cell and parallelness relative to each other by methods described by Ueda et al. (2010). The results of quantification confirmed that there was no significant difference between the average angles and parallelness (*p* = 0.4 and *p* = 0.9, respectively) of GFP-fABD-GFP-labeled actin filaments of triple mutant and wild type (**Figures 7B,C**).

### Sedimentation of Amyloplasts in *xi1 xi2 xik* Is Impaired

**FIGURE 8:**
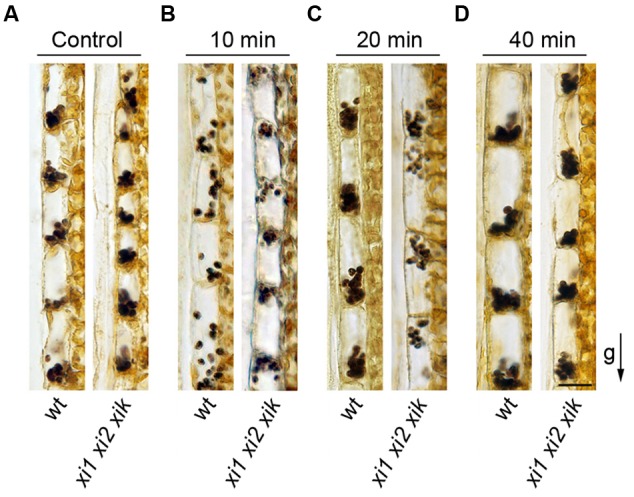
**Amyloplast distribution in endodermal cells. (A)** Localization of amyloplasts in the stems of control plants. **(B–D)** Inflorescence stems were reoriented 180° and gravistimulated for **(B)** 10, **(C)** 20, or **(D)** 40 min. The arrow indicates direction of gravity (g). Scale bar 20 μm.

We then analyzed the localization of amyloplasts in endodermal cells. Stem segments from gravisensitive region were fixed with the direction of gravity maintained constant, sectioned longitudinally and stained with IKI solution. Amyloplasts stained intensely both in wild type and *xi1 xi2 xik* genetic backgrounds indicating that there is no significant difference in the level of starch in amyloplasts. All amyloplasts in wild type and triple mutant control stems located at the bottom side of the cell (**Figure 8A**). Next, we investigated the sedimentation of amyloplasts in the direction of a new gravity vector. Inflorescence stems were gravistimulated for 10, 20, and 40 min by turning stems upside down. After 10 min of reorientation, amyloplasts in wild type stems were localized all over endodermal cell, some of them settling toward the new bottom (**Figure 8B**). Amyloplasts sedimented fully at the bottom of new basal end within 20 min (**Figure 8C**). In contrast, no movement of *xi1 xi2 xik* amyloplasts occurred during the first 10 min of reorientation (**Figure 8B**). First signs of sedimentation were visible by the end of 20 min but most of the amyloplasts still remained in their initial positions (**Figure 8C**) relocating to the new basal end only within 40 min (**Figure 8D**). These results demonstrate that myosins XI1, XI2, and XIK are also required for the proper sedimentation of amyloplasts.

### Gravitropic Response of the *xi1 xi2 xik* Is Rescued by the Expression of YFP-Tagged Myosin XIK

**FIGURE 9:**
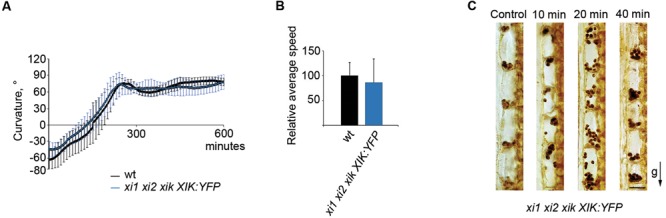
**Effect of YFP-tagged myosin XIK on *xi1 xi2 xik* gravitropism. (A)** Time course of gravitropic response and **(B)** relative average tip movement speed of wild type (wt) and *xi1 xi2 xik XIK:YFP* inflorescence stems. Average speed is expressed as a percentage of wild type average speed (set to 100%). Error bars represent SD, *n* = 7. **(C)** Localization of amyloplasts in *xi1 xi2 xik XIK:YFP* control stems and in 180° reoriented stems gravistimulated for 10, 20, or 40 min. The arrow indicates direction of gravity (g). Scale bar 20 μm.

Despite of the general functional redundancy of the highly expressed myosins XI, it is known that the myosin XIK plays principal roles in such myosin-dependent processes as intracellular trafficking, cell expansion and plant growth (Ojangu et al., 2007, 2012; Peremyslov et al., 2008, 2010, 2012; Avisar et al., 2012; Park and Nebenführ, 2013). Accordingly, we attempted to address this myosin’s contribution to gravitropism using inflorescence stems of *xi1 xi2 xik* triple mutant stably transformed to express a genomic copy of the myosin *XIK* gene tagged with YFP (Peremyslov et al., 2012). Strikingly, the stems of plants with the *xi1 xi2 xik XIK:YFP* genetic background exhibited normal gravitropic response. There was no delay in the gravitropic response of transgenic stems and the curvature was comparable to that of the wild type (**Figure 9A**). Furthermore, the relative average speed of stem movement increased from 26% of that in the wild type in the *xi1 xi2 xik*, to 86% in the *xi1 xi2 xik XIK:YFP* (**Figure 9B**) being not significantly different from that of the wild type (*p* = 0.5). We then investigated the sedimentation of amyloplasts in *xi1 xi2 xik XIK:YFP*, inverting the stems upside down similarly to *xi1 xi2 xik* (**Figure 8**). It was found that translocation of amyloplasts was improved—although 10 min after reorientation amyloplasts in *xi1 xi2 xik XIK:YFP* did not move at the same extent as in wild type, the translocation was massive after 20 min (**Figure 9C**). Thus, the expression of XIK:YFP resulted in genetic rescue of the triple mutant gravitropic bending and amyloplast sedimentation.

## Discussion

In this study, we characterized the roles of all 17 members of the myosin family in the gravitropic response of inflorescence stems of *Arabidopsis*. By using single and multiple mutants of class VIII and class XI myosins we showed that: (1) elimination of one or two myosins does not affect stem gravitropism; (2) simultaneous inactivation of all class VIII myosins does not affect stem gravitropism; (3) simultaneous inactivation of class XI myosins XI1, XI2 and XIK leads to delayed and reduced gravitropic bending correlated with abnormal amyloplast sedimentation, slower growth rate and formation of stiffer inflorescence stem; (4) stable expression of XIK:YFP can restore normal gravitropic response and amyloplast sedimentation.

The fact that gravitropic behavior of all *Arabidopsis* single myosin mutant plants was indistinguishable from wild type was not unexpected. It is well established that myosins have redundant functions and more than one myosin needs to be eliminated for assigning new roles (Prokhnevsky et al., 2008; Peremyslov et al., 2010; Ojangu et al., 2012). It should be mentioned, however, that the polarized growth of root hairs is significantly affected by elimination of either myosin XIK or XI2, making these cells the most sensitive indicator of myosin function (Ojangu et al., 2007; Peremyslov et al., 2008). In our experiments, double mutants *xi1 xi2, xi1 xik, xi2 xik, xih xik* and triple mutant *xi2 xik xih* did not show alterations in the gravitropic response. However, in the study of Okamoto et al. (2015) double mutant *xif xik* exhibited hyperbending of inflorescence stems in response to gravistimulation. According to public *Arabidopsis* eFP browser (Winter et al., 2007), XIF is expressed in the stem at particularly high level. At the same time, myosins XI1, XI2, XIK, and XIH are broadly expressed throughout plant tissues. The fact that *xif xik* showed impaired straightening is more intriguing because it is considered to be a separate process from gravitropic bending (Stanković et al., 1998; Bastien et al., 2013) and amyloplast-mediated gravity sensing (Okamoto et al., 2015). Stem straightening is proposed to be triggered by mechanosensitive channels activated by actin filaments which function as tension sensors in fiber cells (Okamoto et al., 2015). Here we show that *xik* together with *xi1* and *xi2* in triple mutant severely and specifically affect gravitropic bending and there are several reasons for this phenotype.

One important result of this study is that *xi1 xi2 xik* stems had reduced growth rate and significantly increased stiffness in 3-point flexure test compared to the wild type, suggesting a role of myosins in stem architecture. It is possible that stiffness could simply be caused by increased cell number per stem volume due to decreased cell size (Peremyslov et al., 2010; Ojangu et al., 2012). The more cell wall, the stiffer the inflorescence. It has also been reported that cellulose microfibril angles are important in determining cell wall thickness and that microfibril structure and orientation are changed during gravitropism (Folsom and Brown, 1987; Burk and Ye, 2002). Thus, already stiffer inflorescence stem may affect these processes and alter bending response of the stem. Together with stiffness, reduced growth rate of triple mutant found in this study also affects bending. *Shoot gravitropism* mutant *sgr1* exhibited thinner and shorter inflorescence stems, reduced growth rate and failed to curve upward. However, *sgr1* was later found to lack normal endodermal cell layer, essential for gravitropism, in hypocotyls and stems (Fukaki et al., 1996, 1998). Similarly, tropic defects of *grv2* hypocotyls were also associated with reduced growth rate (Silady et al., 2004). For cell elongation, new cell wall components have to be integrated. Trafficking of Golgi-derived secretory vesicles containing cell wall components is proposed to be mediated by actomyosin system and myosin XIK in turn has been shown to contribute to the plant cell physiology by vesicle transport (Nebenführ et al., 1999; Peremyslov et al., 2012). Moreover, cytoplasmic streaming is proposed to be a primary route for organelle and carrier vesicle trafficking driven by myosin-MyoB compartment (Peremyslov et al., 2013, 2015). Therefore, the role of class XI actomyosin system in cell extension during gravitropism would be compatible—in the absence of myosins XI1, XI2, and XIK cell growth is affected and this could reflect in the reduced bending rate of inflorescence stems. In addition, this hypothesis would be consistent with the myosin triple mutant exhibiting overall dwarfing phenotype, including reduced shoot size (Peremyslov et al., 2010; Ojangu et al., 2012). However, growth rate and stiffness of *xi1 xi2 xik* stem are not the only limiting factors in the formation of triple mutant’s gravitropic response.

Another relevant finding was altered sedimentation of amyloplasts in the mutant plants. Dynamic movement of amyloplasts depends on actin filaments (Saito et al., 2005; Nakamura et al., 2011; Zhang et al., 2011). The *sgr9* mutant of *Arabidopsis* exhibited reduced stem gravitropism and amyloplast sedimentation because of denser network of actin filaments enmeshed amyloplasts and reduced their normal sedimentation (Nakamura et al., 2011). In central columella cells of *Arabidopsis* root it was also found that ARP3 mutants display thick actin bundles surrounding amyloplasts that possibly affect amyloplast kinetics, indicating that actin cytoskeleton may regulate amyloplast movement through regulating local viscosity of the cells (Zou et al., 2016). However, the intracellular environments in shoot and root endodermal cells differ considerably (Blancaflor, 2013). Here, we analyzed the organization of actin filaments in living endodermal cells of *xi1 xi2 xik* stems in respect to average angle and parallelness and found no significant changes. This is consistent with the cell type-specific pattern of F-actin reorganization in response to myosin inactivation (Peremyslov et al., 2010; Ueda et al., 2010; Cai et al., 2014). In particular, the epidermal cells of hypocotyls in *xi1 xi2 xik* mutant exhibited less dense F-actin arrays, more bundling and reduction in actin dynamics (Cai et al., 2014). The previous study of the same mutant by Peremyslov et al. (2010) reported reorientation of the F-actin bundles in the leaf midvein epidermal cells but not in the leaf pavement cells or root epidermal cells. Our results showing no apparent changes in F-actin organization in the endodermal cells fit this pattern rather well, although resolution of our analysis could be insufficient to detect more subtle changes in the thin microfilaments that could contribute to F-actin dynamics and amyloplast translocation.

In addition to actin filaments, vacuolar membrane also influences amyloplast dynamics. Vacuolar membrane structures like transvacuolar strands, bulbs, and sheets (Oda et al., 2009) undergo dynamic changes critical in gravity sensing. For example, in *Arabidopsis shoot gravitropism* mutants *sgr2, sgr3, zig/sgr4, sgr6*, and *grv2/sgr8/kam2* amyloplasts localize abnormally and their sedimentation is impaired due to defective vacuolar dynamics (Morita et al., 2002; Yano et al., 2003; Silady et al., 2004; Hashiguchi et al., 2014). Here, the localization of amyloplasts in control plants of wild type and triple mutant was normal. However, Ueda et al. (2010) reported that the development of transvacuolar cytoplasmic strands, ER flow, formation of mobile ER strands, and the configuration of ER network were defective in *xi1 xi2 xik*. Changes in ER flow could influence the overall intracellular dynamics (Stefano et al., 2014) and also affect amyloplast translocation both within and outside transvacuolar strands. It remains to be shown if the ER flow is driven by the ER-associated myosins or follows cytoplasmic streaming.

Furthermore, as mentioned above, the myosin XI function in cytoplasmic streaming may offer even more plausible and simple explanation for myosins’ contributions to gravitropic response. It has been demonstrated that progressive elimination of myosins XI reduces velocities of organelle trafficking that is virtually frozen in the triple mutant *xi1 xi2 xik*, as well as affects cell expansion, suggesting a functional link between these two processes (Peremyslov et al., 2010). Reciprocally, increasing these velocities by using more powerful, engineered myosin XI results in a boost in cell growth thus validating this functional link (Tominaga et al., 2013). Given that the principal driver of organelle and vesicle trafficking and cell growth, myosin XIK, is associated primarily with the novel vesicular compartment defined by myosin receptors termed MyoBs (Peremyslov et al., 2012, 2013), it was suggested that the myosin-MyoB compartment plays a central role in driving both the trafficking and cell expansion. Most recently, it was shown that interference with the activities of myosins and/or their cognate MyoB receptors directly affects cytoplasmic streaming and, to the exact same extent, the organelle and vesicle trafficking (Peremyslov et al., 2015). Taken together, these findings support a model, according to which the myosin-MyoB vesicular compartment drives cytoplasmic streaming, which carries organelles and secretory vesicles thus elevating metabolic status of the cell and aiding cell growth and plant development. An expected corollary of nearly arrested streaming in the *xi1 xi2 xik* would be reduced cytosol hydrodynamics and decelerated amyloplast sedimentation in response to gravity vector. Fittingly, interference with streaming also results in morphogenic defects that include erratic rather than straight and upward stem and silique orientation, as well as “wavy” root appearance (Peremyslov et al., 2015), a phenotype compatible with “confused” gravitropic response.

The phenotype of *xi1 xi2 xik* gravitropic bending and amyloplast sedimentation was rescued by the expression of XIK:YFP. The functional competence of XIK:YFP was previously validated by Peremyslov et al. (2012), who showed that virtually all plant growth defects of triple mutant were rescued by XIK:YFP expression, implying significant functional contributions of myosin XIK. Although it is tempting to speculate that XIK is the main contributor to stem gravitropic response, because XIK was sufficient to complement the gravitropic response related phenotype of the triple mutant, it remains to be confirmed by further experiments.

Conversely to class XI, class VIII myosins were not involved in gravitropism demonstrating different function of these two classes in plants. Indeed, knocking out *Arabidopsis* class VIII myosins one by one and all together did not affect stem gravitropic response. Also, *viii1 viii2 viiia viiib* mutant did not exhibit obvious developmental defects and its overall growth phenotype was similar to that of the wild type (data not shown). Analysis of loss-of-function mutant of all five myosin VIII of *Physcomitrella patens* revealed their role in development, hormone homeostasis and in phragmoplast expansion (Wu et al., 2011; Wu and Bezanilla, 2014), whereas no effects in moss gravitropism were reported.

In conclusion, our results support the idea that myosins XI1, XI2, and XIK are involved in stem gravitropism. Amyloplast sedimentation and physical features of the stem are important in formation of gravitropic response and we showed that in myosin triple mutant both of them are affected. Whether these myosins contribute to gravitropism through shaping actin cytoskeleton, via driving microfilament sliding and thereby mediating repositioning of amyloplasts, or via reorganization and streaming of the ER, remains to be elucidated. However, the most parsimonious mechanistic explanation of the observed effects is myosin involvement in driving cytoplasmic streaming and thus increasing both the cytosol fluidity, amyloplast sedimentation and cell growth. Through these different cellular processes, myosin XI is crucial in gravity-induced bending of *Arabidopsis* stems.

## Author Contributions

HP conceived the study; HP, BI, and KTal conducted experiments and analyzed data. KTal, BI, HP, ET, and VD wrote the manuscript; E-LO generated *xi1 xi2 xik*; KTan generated *xi2 xik xih* mutant; and VP generated myosin VIII mutants and contributed to writing. All authors read and approved the manuscript.

## Conflict of Interest Statement

The authors declare that the research was conducted in the absence of any commercial or financial relationships that could be construed as a potential conflict of interest.
