# Supplementary material for: Arabidopsis Myosins XI1, XI2, and XIK Are Crucial for Gravity-Induced Bending of Inflorescence Stems
Source: Front Plant Sci. 2016 Dec 21;7:1932. doi: 10.3389/fpls.2016.01932 (PMC5174092; doi:10.3389/fpls.2016.01932)
Supplement: Supplementary file 1 [file Table_1.DOCX]

Supplementary Material

*Arabidopsis* myosins XI1, XI2 and XIK are crucial for gravity-induced bending of inflorescence stems

Kristiina Talts*, Birger Ilau, Eve-Ly Ojangu, Krista Tanner, Valera V. Peremyslov, Valerian V. Dolja, Erkki Truve, Heiti Paves

*** Correspondence:** Kristiina Talts: krezzu@gmail.com

# Supplementary Figures and Tables

Supplementary Table S1. *Arabidopsis thaliana* T-DNA lines.

| Name | Insertion line | Alternative name | Reference |
| --- | --- | --- | --- |
| *viii1* | Sail_405_B08 |  | Haraguchi et al., 2014 |
| *viii1(1)* | Salk_022962 |  |  |
| *viii2* | Salk_052429 |  |  |
| *viiia* | Sail_1307_G01 |  |  |
| *viiia(1)* | Salk_062355 |  |  |
| *viiib* | Salk_009323 |  |  |
| *viiib(1)* | Salk_134493 |  |  |
| *xi1* | Salk_022140 | *xi1-2* | Ueda et al., 2010; Ojangu et al., 2012; Okamoto et al., 2015 |
| *xi1(1)* | Salk_019031 | *xi1* | Peremyslov et al., 2008 |
| *xi2* | Sail_632_D12 |  | Peremyslov et al., 2008; Ojangu et al., 2012 |
| *xi2(1)* | Salk_127984 | *xi2-1* | Ueda et al., 2010 |
| *xia* | Salk_145017 |  |  |
| *xib* | Salk_113062 | *xib-1* | Peremyslov et al., 2008; Madison et al., 2015; Okamoto et al., 2015 |
| *xic* | Sail_905_C08 | *xic-2* | Madison et al., 2015 |
| *xid* | Salk_029987 |  |  |
| *xie* | Salk_119881 |  |  |
| *xif* | GK-401H02 |  |  |
| *xig* | Salk_018032 | *xig-1* | Peremyslov et al., 2008; Okamoto et al., 2015 |
| *xih* | Salk_014709 |  |  |
| *xii* | Salk_082443 |  | Peremyslov et al., 2008 |
| *xij* | Salk_063159 |  | Peremyslov et al., 2008 |
| *xik* | Salk_067972 | *xik-2* | Ojangu et al., 2007; Peremyslov et al., 2008 |
| *xik(1)* | Salk_136682 | *xik-1* | Ojangu et al., 2007; Ueda et al., 2010; Okamoto et al., 2015 |
