# Supplementary material for: Arabidopsis Myosins XI1, XI2, and XIK Are Crucial for Gravity-Induced Bending of Inflorescence Stems
Source: Front Plant Sci. 2016 Dec 21;7:1932. doi: 10.3389/fpls.2016.01932 (PMC5174092; doi:10.3389/fpls.2016.01932)
Supplement: Supplementary file 2 [file Table_2.DOCX]

Supplementary Material

*Arabidopsis* myosins XI1, XI2 and XIK are crucial for gravity-induced bending of inflorescence stems

Kristiina Talts*, Birger Ilau, Eve-Ly Ojangu, Krista Tanner, Valera V. Peremyslov, Valerian V. Dolja, Erkki Truve, Heiti Paves

*** Correspondence:** Kristiina Talts: krezzu@gmail.com

# Supplementary Figures and Tables

Supplementary Table S2. RT-qPCR primers.

| Gene name | AGI code | Primer sequence |
| --- | --- | --- |
| SAND | AT2G28390 | 5’ - AACTCTATGCAGCATTTGATCCACT - 3’ |
|  |  | 5’ - TGATTGCATATCTTTATCGCCATC - 3’ |
| UBC | AT5G25760 | 5’ - CTGCGACTCAGGGAATCTTCTAA - 3’ |
|  |  | 5’ - TTGTGCCATTGAATTGAACCC - 3’ |
| expressed sequence (EX70) | AT2G32170 | 5’ - ATCGAGCTAAGTTTGGAGGATGTAA - 3’ |
|  |  | 5’ - TCTCGATCACAAACCCAAAATG - 3’ |
| PP2A subunit PDF2 | AT1G13320 | 5’ - TAACGTGGCCAAAATGATGC - 3’ |
|  |  | 5’ - GTTCTCCACAACCGCTTGGT - 3’ |
| VIII1 | AT3G19960 | 5’ - GTCGTTTGCTAATTAAGGATTTGG - 3’ |
|  |  | 5’ - TTAACATTGAGAGGCAAATACGAG - 3’ |
| VIII2 | AT5G54280 | 5’ - CGGAAGTTTGAATGCGGTTAAT - 3’ |
|  |  | 5’ - CGAGCTTCACCTCCACAATAG - 3’ |
| VIIIA | AT1G50360 | 5’ - AGAGTCATGTTTTTCATTTGCCAC - 3’ |
|  |  | 5’ - CAAATACAGCAATGGTTCACTGG - 3’ |
| VIIIB | AT4G27370 | 5’ - AAGTCTTGTCGAGGTGAAATCAGA - 3’ |
|  |  | 5’ - CTTCATCACCATTTAACCGGACTC - 3’ |
| XI1 | AT1G17580 | 5’ - CGCTAGGACATTTTCAGATTAGGA - 3’ |
|  |  | 5’ - TAGAACAGTACCAGAAAGCCACT - 3’ |
| XI2 | AT5G43900 | 5’ - GGAATCAAACCAGCAGAAGAAC - 3’ |
|  |  | 5’ - ACAATCACAGAGGAAGAGAGC - 3’ |
| XIA | AT1G04600 | 5’ - TGTTTCGGCCTTGGTATAACATG - 3’ |
|  |  | 5’ - ACTGCCTTTTAGGGTTTCTTATCC - 3’ |
| XIB | AT1G04160 | 5’ - ATTCCCTTCTCAATAGACGAGATTT - 3’ |
|  |  | 5’ - ATACGAATTCTGGGTTTTCCAGTA - 3’ |
| XIC | AT1G08730 | 5’ – ATCGAACCGCCACCTTTAATC - 3’ |
|  |  | 5’ - TCCGTTGGTGTGTGTGTATTG - 3’ |
| XID | AT2G33240 | 5’ - CCCTTTGCAGCTGATGAAATC - 3’ |
|  |  | 5’ - TCCTTCAGAAAGTGGAAGTTAGG - 3’ |
| XIE | AT1G54560 | 5’ - ATCGGGTTTCTGGTCATACAC - 3’ |
|  |  | 5’ - CTGGTGATACGCTATGAGTTCC - 3’ |
| XIF | AT2G31900 | 5’ - CTCACATCAAAAGACACAGAAATCA - 3’ |
|  |  | 5’ - TAGTCCCGATTCGATATACTTGTG - 3’ |
| XIG | AT2G20290 | 5’ - GTTCAATGCAAGATAAGGACTTTG - 3’ |
|  |  | 5’ - CTGAGATTCTTCTTCATGCTTTTG - 3’ |
| XIH | AT4G28710 | 5’ - GTACTGTCCCTTCAAGAACACAC - 3’ |
|  |  | 5’ - CATAGCATAGACCTTAAGGAACTTC - 3’ |
| XII | AT4G33200 | 5’ - TTGAACGATTGAAGCTTAGACTTG - 3’ |
|  |  | 5’ - GTCCAAACAAAAGAGAAAACTGACA - 3’ |
| XIJ | AT3G58160 | 5’ - CATCTCCTTCCTCAGACAATTTTT - 3’ |
|  |  | 5’ - TGTCGTAGCTCTCAGTTCCTTT - 3’ |
| XIK | AT5G20490 | 5’ - GTGGAGAGGCCGAGTTTATTT - 3’ |
|  |  | 5’ - GACAGCATCAAACACACCTAAAG - 3’ |
